# Fear of Falling among Community-dwelling Sedentary and Active Older People

**DOI:** 10.17533/udea.iee.v39n1e13

**Published:** 2021-03-05

**Authors:** Alejandra-Ximena Araya, Evelyn Iriarte

**Affiliations:** 1 Nurse-midwife, PhD. Universidad Andrés Bello, Santiago, Chile. E-mail: alejandra.araya.g@unab.cl. Corresponding author Universidad Andrés Bello Universidad Andrés Bello Santiago Chile alejandra.araya.g@unab.cl; 2 Nurse, PhD. Pontificia Universidad Católica de Chile, Santiago, Chile. University of Miami, Miami, USA. E-mail: esiriart@uc.cl Pontificia Universidad Católica de Chile Pontificia Universidad Católica de Chile Santiago Chile esiriart@uc.cl

**Keywords:** accidental falls, aged, geriatric assessment, depression, fear, exercise, accidentes por caídas, anciano, evaluación geriátrica, depresión, miedo, ejercicio, acidentes por quedas, idoso, avaliação geriátrica, depressão, medo, exercício

## Abstract

**Objective.:**

The study sought to compare community-dwelling older people with respect to their level of physical activity and to the fear of falls between a group of sedentary elderly and a group of active elderly.

**Methods.:**

Cross-sectional descriptive study carried out with 113 community-dwelling older people (45 sedentary and 48 active), users of an outpatient care center of the private health system with a geriatric program in Santiago, Chile. The study measured socio-demographic variables, state of health, comprehensive geriatric assessment, exercise, depression with the Yesavage scale, and fear of falling with the *Short Falls Efficacy Scale - International* (Short FES-I).

**Results.:**

Sedentary older people have significantly higher scores in the Yesavage depression scale compared with active older people (4.2 versus 0.8). No statistically significant differences were found when comparing both groups of sedentary and active participants in terms of socio-demographic variables along with health, and functional and cognitive capacity. Regarding the fear of falling, the sedentary had a slightly higher score than the active (12 versus 11), although not significant.

**Conclusion.:**

This study showed that fear of falling was equal in sedentary and active older people who live in the community, although it was found that sedentary individuals had a higher risk of having a positive screening for geriatric depression in those participants who do not perform physical activity.

## Resumen

**Objetivo.:**

Comparar las personas mayores (PM) de un grupo de mayores sedentarios y otro no sedentario con respecto a su nivel de actividad física y al temor a las caídas.

**Métodos.:**

Se trata de un estudio descriptivo de corte transversal, realizado en 113 personas mayores (45 sedentarios y 48 activos) que viven en la comunidad de usuarios de un centro de atención ambulatoria del sistema privado de salud, el cual disponía de un programa de geriatría en Santiago de Chile. Se midieron variables sociodemográficas, de estado de salud, de Valoración Geriátrica Integral, ejercicio, la depresión con escala de Yesavage y el temor a caer con el *Short Falls Efficacy Scale - International* (Short FES-I).

**Resultados.:**

Las PM sedentarias presentan puntuaciones significativamente más altas en la escala de depresión Yesavage comparadas con las PM activas (4.2 versus 0.8). No se encontraron diferencias estadísticamente significativas al comparar ambos grupos en cuanto a variables sociodemográficas, de salud y de capacidad funcional y cognitiva. En cuanto al temor a caer, los sedentarios tuvieron un puntaje ligeramente mayor que los activos (12 versus 11), aunque sin ser significante.

**Conclusión.:**

Este estudio mostró que el temor a caer fue igual en PM sedentarias y activas, aunque se encontró un mayor riesgo de tener un tamizaje positivo para depresión geriátrica en aquellos participantes que no realizan actividad física.

## Resumo

**Objetivo.:**

Comparar idosos (PM) que vivem na comunidade em relação ao nível de atividade física e medo de cair entre um grupo de idosos sedentários e outro.

**Métodos.:**

Estudo descritivo transversal realizado com 113 idosos residentes na comunidade (45 sedentários e 48 ativos) usuários de um centro de atenção ambulatorial do sistema privado de saúde com programa geriátrico em Santiago, Chile. Variáveis ​​sociodemográficas, estado de saúde, Avaliação Geriátrica Abrangente, exercício, depressão com a escala de Yesavage e medo de cair foram mensurados com a *Short Falls Efficacy Scale - International (Short FES-I).*

**Resultados.:**

PMs sedentários têm pontuações significativamente mais altas na escala de depressão de Yesavage em comparação com PMs ativos (4.2 versus 0.8). Não foram encontradas diferenças estatisticamente significativas ao comparar os dois grupos de participantes sedentários e ativos em termos de variáveis ​​sociodemográficas, saúde e capacidade funcional e cognitiva. Em relação ao medo de cair, os sedentários tiveram uma pontuação ligeiramente superior aos ativos (12 versus 11), embora não tenha sido significativa.

**Conclusão.:**

Este estudo mostrou que o medo de cair era o mesmo em PM sedentários e ativos que viviam na comunidade, embora pessoas sedentárias tenham um risco maior de ter uma triagem positiva para depressão geriátrica naqueles participantes que não realizavam atividade física.

## Introduction

Fear of falling is one of the biggest worries among older people and should not be underestimated. This term has been conceptualized as the confidence a person has to carry out activities, losing balance, or falling.(1) Fear of falling is one of the principal predictors of future falls among the elderly population.(2^,^^3^) It has been estimated that the prevalence of the fear of falling is close to 65% among the elderly without prior falls, rising to 90% in those with antecedents of falls.(4) A higher number of comorbidities, low level of physical activity, worse performance in activities of daily life, and restriction in mobility have been described as predictors of fear of falling among older people.(5)

Fear of falling is not only a frequent problem among community-dwelling older individuals but is also recognized as an important public health problem.(6) Falls and the fear of falling can cause critical physical and psychological changes, like physical self-limitation and dependence in the elderly.(5) This phenomenon can trigger diminished mobility and independence, as well as disability, leading to a loss of confidence, restriction of physical activities, and social participation.(1^,^^6^^,^^7^) Latin American studies have identified that being a woman, 75 years old or over, with alterations in static balance in standing position, dizziness or vertigo, poor or very poor self-perception of health, and movement alterations increase the risk of fear of falling significantly.(8^,^^9^) Upon being associated with restriction of activity, as well as worsened physical and cognitive functions, fear of falling contributes to an important decrease in quality of life among those with antecedents of prior falls, as well as among those without such antecedents.(10) Besides, fear of falling has been associated with increased care and costs related with health and institutionalization, leading - finally - to premature mortality.(1^,^^6^)

Physical exercise has proven to be an effective strategy to diminish the fear of falling and falls in the elderly population.(11^,^^12^) Kendrick *et al.,* indicate that physical exercise, as strategy to prevent falls, reduces the fear of falling after the intervention, without increasing the number of falls of the participants.(12) Given the importance of physical exercise and its implications in preventing the fear of falling, the aim of this study was to compare older people (OP) who live in the community with respect to their level of physical activity and the fear of falling between a group of sedentary elderly and a group of active elderly.

## Methods

Design. Cross-sectional descriptive study, with a sample of 113 older adults who were users of an outpatient care center of the private health system, which had a geriatric program in Santiago, Chile. Inclusion criteria were: being over 60 years of age and without prior history of fractured hip, and without medical diagnosis of cognitive impairment. The study excluded those with diagnosed dementia and who were unable to answer the survey. The study included all the individuals who attended medical control during the second semester of 2013 and complied with the criteria described. The categories of physical activity among the participants were established in accordance with the recommendations by the World Health Organization (WHO), defining as *active* person whomever dedicated over 150 min per week to performing moderate aerobic physical activities, and *sedentary* if they did not comply with the definition by the same organization.(13) Based on the aforementioned, two groups were defined: sedentary (*n* = 45) and active *(n* = 68).

Data collection. The participants were interviewed by research assistants trained in data collection. Application of the questionnaire took nearly 45 min. The questionnaire designed contained: (i) socio-demographic characteristics: sex, age, marital status, educational level, employment situation, if living accompanied, and if they had sons or daughters; (ii) characteristics of health status: having a chronic disease, taking medications regularly, perception of health, and satisfaction with life in the last six months; and (iii) comprehensive geriatric assessment: this measured variables of *Functional capacity* referring to the performance of activities of daily life (ADL) through Barthel's scale, with a score from 0 to100, with the highest score indicating greater independence.(14) To measure the instrumental activities of daily life (IADL), the study used Lawton's scale, where 0 points represents dependence and 8 points total independence;(15) *Cognitive and affective capacity*- for the cognitive state the work used the Mini-mental State Examination (MMSE) by Folstein to screen dementia in its version adapted by age and education level to identify cognitive impairment.(16) This scale has a total score range from 0 to 30, where the highest scores indicate better cognitive function. Depression symptoms were measured through the 15-item Yesavage scale (Yes/No), where a score of 6 or more indicates possible screening of depression.(17)

(iv) Fear of falling was measured through the *Short Falls Efficacy Scale - International* (Short FES-I). The short version FES-I has seven items with Likert scale with four categories, including the options "Not at all concerned = 1", “Somewhat concerned = 2”, “Fairly concerned = 3”, and “Very concerned = 4” of falling during activities of their daily life. The scoring system ranges from 7 to 28 points. The highest values indicate greater fear of falling.(18) The FES-I has shown to have adequate psychometric properties in different populations of OP (18) including Chilean population.(19)

Data analysis. A descriptive analysis of means, medians, percentiles, standard deviations, absolute and relative frequencies was carried out for quantitative variables; and percentages for the nominal variables. Comparisons were made between the active and sedentary groups with Student’s t test for independent samples and Chi squared for dichotomous variables, considering a significance < 0.05. The IBM SPSS 25.0 program was used for data analysis.

Ethical Aspects. This research adhered to the ethical standards of the World Medical Association and the Helsinki declaration. This study was approved by the Ethics Committee of the School of Nursing at Pontificia Universidad Católica de Chile.

## Results

For the population studied (*n*=113), the mean age was 70.8±6.9 years; 80.5% (*n*=91) were women, 56.6% (*n*=64) married, 76.1% were retired (*n*=86), and 82.3% (*n*=93) lived accompanied with a spouse and/or sons or daughters. No participant was reported as illiterate. Concerning the characteristics of health status, most participants classified their health status as good and very good, represented by 59.2% (*n*=67); 80.5% (*n*=91) declare having at least one chronic disease and 71.7% (*n*=81) regularly takes at least one medication per day; 60.2% (*n*=68) report performing physical exercise according to recommendations by the WHO, while the rest define themselves as sedentary. Table 1 shows the socio-demographic characteristics and of the state of health according to study group. No significant differences existed between both study groups.

**Table 1:** Characterization of the socio-demographic and health status variables of the community-dwelling older people, according to study group

Variables	Group	Group	***p-*value**
Variables	Sedentary (*n*=45)	Active (*n*=68)	***p-*value**
Socio-demographic characteristics			
Age; mean ± SD	70.6±6.9	70.9±6.9	0.573
Sex: Female; *n* (%)	40 (88.9)	51 (75.0)	0.068
Has children; *n* (%)	41 (9.1)	55 (80.9)	0.137
Lives accompanied; *n* (%)	37 (82.2)	56 (82.4)	0.978
Years of education; average ± SD	11.3±4.8	11.6±4.5	0.650
Characteristics of health status			
Self-perception of health *n* (%)			
Excellent/Very good	5 (11.2)	11 (16.2)	0.055
Good	20 (44.4	37 (54.4)	0.345
Poor/Very poor	20 (44.4)	20 (29.4)	
Satisfaction with life *n* (%)			
Very satisfied/Satisfied	30 (66.7)	53 (77.9)	
Poorly satisfied/Dissatisfied	15 (33.3)	15 (22.1)	

Table 2 presents the differences in the comprehensive geriatric assessment characteristics and fear of falling according to the physical exercise classification of the study sample. Regarding Barthel’s index and the Lawton and Brody scale, most of the participants in this study are independent for activities of daily life (80%, *n* = 91) and instrumental activities (74.0%; *n* = 83), without cognitive impairment, with an average of 28±1.9 in the MMSE and with negative depression screening (73.0%; *n* = 82), with a mean of 3.5±3.2 in the Yesavage depression scale (maximum of 15 points). When comparing by groups, the group of sedentary individuals has significantly higher scores in the Yesavage depression scale compared with the group of active individuals. The rest of the variables studied do not show statistically significant differences. According to the short FES-I scale, sedentary individuals got 12 points versus 11 points for the active individuals, without this being a statistically significant difference between both study groups.

**Table 2:** Comprehensive geriatric assessment and fear of falling of the community-dwelling older people, according to study group

Measurement scales	Group	Group	***p*-value**
Measurement scales	Sedentary (*n*=45)	Active (*n*=68)	***p*-value**
Functional capacity			
Barthel’s index; average ± SD	98.0±4.2	98.8±4.1	0.142
Lawton and Brody scale; average ± SD	7.5±0.9	7.6±0.8	0.205
Cognitive and affective capacity			
Yesavage scale (Depression); average ± SD	4.2±3.8	0.8±0.4	0.007
MMSE (Cognition); average ± SD	28.4±1.7	27.8±2.0	0.105
Fear of falling			
Short FES-I; average ± SD	12.0±5.1	11.0±4.0	0.275

Table 3 includes the frequency of each item characterized according to study group. Going up or down stairs, reaching for something above your head or on the ground, and walking up or down a slope are the activities that generate the most fear of falling, both in active and sedentary OP. No significant differences were found between both groups for the variables of this scale.

**Table 3:** Percentage of adults who are very concerned about falling, according to the items from the FES-S instrument according to study group

Variable	Group	Group
Variable	Sedentary (*n*=45)	Active (*n*=68)
Getting dressed or undressed; *n* (%)	4 (8.9)	4 (5.9)
Taking a bath or shower; *n* (%)	8 (17.8)	10 (14.7)
Getting in or out of a chair	2 (4.0)	2 (2.9)
Going up or down stairs; *n* (%)	14 (31.1)	16 (23.5)
Reaching for something above your head or on the ground; *n* (%)	14 (31.1)	17 (25.0)
Walking up or down a slope; *n* (%)	13 (28.9)	19 (27.9)
Going out to a social event; *n* (%)	3 (6.7)	4 (5.9)

## Discussion

The aim of this study was to compare indicators of comprehensive geriatric assessment (functional, cognitive, and affective capacity) and of fear of falling among older sedentary and active persons. Although the literature is robust in supporting that physical exercise is an effective strategy to diminish fear of falling,(11^,^^12^) this study found no statistically significant differences between sedentary and active people. Differences were only identified among the scores for geriatric depression screening, with greater risk of having a positive screening for depression in participants who do not engage in physical activity.

In accordance with the results of this study, it is fitting to wonder if fear of falling is one of the causes for the elderly to avoid practicing physical activity. Prior studies have described that fear of falling is an important barrier for older persons to perform physical activity.(20^,^^21^) However, Tam-Seto *et al*., identified a series of other factors that would discourage participation in physical activities, finding that lack of motivation, lack of companionship, and lack of access were relevant factors to consider.(22) It is important to reinforce recruitment aspects for older people to adhere to the type of physical exercise they choose to keep active. Moreover, this study considered the recommendations by the WHO to differentiate between sedentary and active people. However, the WHO defines as active person that older adult engaged in over 150 min per week to performing moderate aerobic physical activities and a series of recommendations that can vary according to the health status of those conducting them.(13)

This study showed that active participants had lower risk of positive screening for geriatric depression compared with those who are sedentary. The aforementioned agrees with that reported in the literature concerning exercise in high dosage is associated with improvement in the mental and physical domains of quality of life.(23-25) Given that geriatric depression has been associated with greater fear of falling,(20) future studies should focus on the relationship that exists among these three variables: fear of falling, depression, and performance of physical activity. Our study had a series of limitations. First, the participants in this study were users of a single health center, without functional impairment, dementia diagnosis or depression, which does not necessarily represent the health of older Chilean people. Additionally, this sample reported a high level of physical activity, unlike that reported nationally, where over 80% of the older adult population define themselves as sedentary in the last three months. Another important limitation is the sample size, which could cause differences between the groups of active and sedentary participants to not be statistically significant. It is recommended to perform studies contemplating a greater sample size per group and including differentiation among types of exercises and their frequency.

An explanation for not having found significant differences between active and sedentary groups with respect to fear of falling could be explained by the criteria used to define the distinct groups. This study used the definition by the WHO to classify older individuals between active or sedentary. That definition focuses on the number of minutes during which the older person engages in exercise and not on the type of exercise conducted. Further research should focus on evaluating the impact of fear of falling on performing physical exercise, discriminating by its type, frequency, and intensity. 
